# A First Report of *Aeromonas veronii* Infection of the Sea Bass, *Lateolabrax maculatus* in China

**DOI:** 10.3389/fvets.2020.600587

**Published:** 2021-01-20

**Authors:** Baotun Wang, Can Mao, Juan Feng, Yong Li, Jianmei Hu, Biao Jiang, Qunhong Gu, Youlu Su

**Affiliations:** ^1^Innovative Institute of Animal Healthy Breeding, Zhongkai University of Agriculture and Engineering, Guangzhou, China; ^2^Key Laboratory of South China Sea Fishery Resources Development and Utilization, Ministry of Agriculture and Rural Affairs, Guangzhou, China; ^3^National Demonstration Center for Experimental Fisheries Science Education, Shanghai Ocean University, Shanghai, China; ^4^Modern Agricultural Development Center of Zhuhai City, Zhuhai, China

**Keywords:** *Aeromonas veronii*, *Lateolabrax maculatus*, physiological and biochemical characteristics, pathogenicity, pathology

## Abstract

The sea bass, *Lateolabrax maculatus* is commercially farmed in Zhuhai, located in the Guangdong Province of China. *L. maculatus* in aquaculture have suffered acute death, characterized by ulcerations on the body surface, congestion, and hemorrhage in internal organs such as liver, kidney, and spleen. The dominant infecting strain of bacteria isolated from the kidneys of diseased fish was identified as *Aeromonas veronii* (strain 18BJ181). This identification was based on analysis of morphological, physiological, and biochemical features, as well as 16S rRNA and *gyrB* gene sequences. Drug sensitivity testing showed that the strain 18BJ181 isolate was resistant to four antibacterial drugs, including amoxicillin, madinomycin, penicillin and sulfamethoxazole, while moderately sensitive to erythromycin and rifampicin. The detection of growth characteristics showed that the strain 18BJ181 exhibited adaptability to the environment. In addition, some virulence genes, such as *aer, act, gcaT, tapA* and *fla*, were detected in the strain 18BJ181. The median lethal dosage of the strain 18BJ181 isolate in *L. maculatus* was 8.5 × 10^5^ and 4.2 × 10^5^ cfu/g under the conditions of intraperitoneal injection and intramuscular injection, respectively. The experimentally induced infection showed that the 18BJ181 isolate caused considerable histological lesions in *L. maculatus*, including tissue degeneration, necrosis, and different degrees of hemorrhage. These results provided evidence for a more comprehensive understanding of *A. veronii* strain 18BJ181 infection in *L. maculatus*.

## Introduction

*Aeromonas veronii*, a Gram-negative bacterial pathogen, has a wide range of hosts and can cause diarrhea and sepsis in humans (1). In particular, *A. veronii* is a common pathogen in aquaculture, which can infect a variety of aquatic animals, including freshwater goldfish (*Carassius auratus*) (2), Nile tilapia (*Oreochromis niloticus*,) (3), Chinese Longsnout catfish (*Leiocassis longirostris günther*) (4) and catfish (*Ictalurus punctatus*,) (5, 6). The clinical symptoms of infected fish are skin ulcers and visceral hemorrhage. The histopathological changes caused by *A. veronii* are manifested as cerebral vascular hyperemia, inflammatory cell infiltration, osteoporosis, renal tubular necrosis, and hepatocyte degeneration (7). The virulence of *A. veronii* was shown to be stronger than *Aermonas hydrophila*, which could cause septicemia in fish (8). At present, *A. veronii* is the primary pathogen isolated from freshwater fish in South China.

The sea bass, *Lateolabrax maculatus*, is an economically important, cultured species in East Asia and is an important aquaculture fish in China in particular (9). Viral and bacterial diseases can cause significant damage to cultured *L. maculatus* (10, 11). In 2018, a continuous epidemiological investigation was carried out in the *L. maculatus* culture area in the Zhuhai, Guangdong Province in China, and found that *A. veronii* was an important pathogen. In this study, the results of isolation, identification, drug sensitivity, growing characteristics, virulence gene distribution and pathogenicity of the *A. veronii* isolate are described.

## Materials and Methods

### Sampling of Diseased Fish and Isolation of Bacteria

Diseased *L. maculatus* were sampled from a freshwater fish farm in Zhuhai, Guangdong Province, China. Moribund fish were taken from the pond to a laboratory at the Modern Agricultural Development Center of Zhuhai City, Zhuhai, Guangdong Province, China. Only diseased fish with typical clinical symptoms were used for bacterial examination. The fish were dissected after their skin was cleaned with 75% ethyl alcohol. Liver, spleen, and kidney were used for bacterial isolation. A Nutrient Agar medium (NA) was employed for bacteria isolation for 24 h at 28°C and the dominant uniform bacterial colonies were purified by streaking onto the NA plates twice. A single bacterial colony was selected and inoculated in nutrient broth (NB) for 14 h at 28°C, then preserved at −80°C in the NB medium containing 20% (v/v) sterile glycerol. A dominant strain was tentatively named 18BJ181.

### Analysis of Physiological and Biochemical Characterization

Thirty-eight biochemical reactions were performed using Vitek 2 Compact (Biomerieux, France) according to the manufacturer's instructions. Identification results of bacterial species were generated based on the combination of biochemical activities.

### Sequence Analysis of 16S rRNA and *gyrB* Gene

The genomic DNA of the strain 18BJ181 was extracted using a TIANamp Bacterial DNA Kit (Tiangen-Biotech, Beijing, China) following the manufacturer's guidelines. Genomic DNA was stored at −20°C. A pair of universal primers, 8 F:5′- AGAGTTTGATCCTGGCTCAG-3′ and 1492 R: 5′-GGTTACCTTGTTACGACTT-3′, was used for amplification of the 16S rRNA gene. A pair of primers, 3 F:5′-TCCGGCGGTCTGCACGGCGT-3′ and 14 R: 5′-TTGTCCGGG TTGTACTCGTC-3′, was used for amplification of the *gyrB* gene. The mixtures were incubated in a cycle of 95°C for 5 min, followed by 30 cycles of 95°C for 15 s, 55°C for 15 s, and 72°C for 15 s, and extension at 72°C for 10 min. The amplified products were observed and sequenced, then were sent to Guangzhou Tian Yihui Gene Technology Co., Ltd. A BLAST search for sequences was carried out via the NCBI website (https://www.ncbi.nlm.nih.gov/). The phylogenetic trees were established using the Neighbor-joining method in the MEGA 5.1 software package (12).

### Growing Characteristics

The pH value and NaCl concentration were adjusted based on the NB medium. Before sterilization by autoclaving, the pH was adjusted to the desired values with NaOH (1 mol/L) or HCl (1 mol/L). The target salinity values were obtained by adding NaCl to NB. The isolate cultured in NB was incubated at 28°C with pH value of 3, 5, 7, 9, and 11 to evaluate growth characteristics. Similarly, the growth characteristics of the isolate was evaluated in NB at 28°C with a salinity of 5, 10, 20, 40, and 80 ppt, respectively. All flasks were inoculated with 0.2 mL of bacterial suspension (optical density, OD_600_= 0.1) and cultured at 180 rpm in 96-well plates. Growth was monitored for 23 h by measuring the OD with a micrometer at 600 nm every 1 h.

### Antimicrobial Resistance Test

The antibiotic resistance of the strain 18BJ181 was determined by the disk diffusion method [K-B method (13)]. The strain 18BJ181 was cultured in Mueller-Hinton medium and the concentration of the bacterial solution was adjusted to 1 × 10^8^ cfu/ml. The suspension was spread on Mueller-Hinton agar containing either trimethoprim, amoxicillin, chloramphenicol, doxycycline, erythromycin, enrofloxacin, florfenicol, furazolidone, gentamicin, madinomycin, neomycin, norfloxacin, oxytetracycline, penicillin, rifampicin or sulfamethoxazole (all purchased from Hangzhou microbial Reagent Co. Ltd). According to the size of the bacteriostatic zone, the results of drug sensitivity were judged by sensitivity, mediating, and drug resistance.

### Virulence Genes Detection

Conventional PCR assays for the amplification of the aerolysin (*aer*), cytotoxic enterotoxin (*act*), heat-stable enterotoxin (*ast*), glycerophospholipid-cholesterol acyltransferase (*gcaT*), extracellular deoxyribonuclease (*exu*), Type IV pilus (*tapA*), *lip* and flagellin (*fla*) were performed with the template DNA of the strain 18BJ181. Primers used for amplification of the eight genes are shown in Table 1. Each PCR reaction contained 12.5 μl of PCR Mix (Tiangen Biotech, Beijing Co., Ltd., China), 1μl of each paired primer, 1 μl of template DNA, and 9.5 μl of ddH_2_O. The PCR reaction commenced with denaturation at 94°C for 2 min, then 35 cycles of amplification, and finally extension at 72°C for 10 min. Each cycle consisted of denaturation at 94°C for 30 s, annealing for 50 s and extension at 72°C for 30 s (Table 1). The amplified PCR products were maintained at 4°C. The results were recorded after electrophoresis on 2% agarose gel stained with ethidium bromide and the positive product of PCR were sent to were sent to Guangzhou Tian Yihui Gene Technology Co., Ltd for analysis and verification (14, 15).

**Table 1:** The information for the eight virulence genes pairs of primers.

**Target gene**	**Primer sequence (5^**′**^-3^**′**^)**	**Product size (bp)**	**T_**m**_ (^**°**^C)**
*aer*	F: TCTCCATGCTTCCCTTCCACT R: CCAGTTCCAGTCCCACCACT	431	63
*act*	F: AGAAGGTGACCACCACCAAGAACA R: AACTGACATCGGCCTTGAACTC	232	65
*ast*	F: TCTCCATGCTTCCCTTCCACT R: GTGTAGGGATTGAAGAAGCCG	331	63
*gcaT*	F: CTCCTGGAATCCCAAGTATCAG R: GGCAGGTTGAACAGCAGTATCT	237	65
*exu*	F: AGACATGCACAACCTCTTCC R: GATTGGTATTGCCTTGCAAG	323	61
*tapA*	F: ATGACCTCTAGCCCCAATA R: ACCCGATTGATTTCTGCC	550	55
*lip*	F: ATCTTCTCCGACTGGTTCGG R: CCGTGCCAGGACTGGGTCTT	382	63
*fla*	F: TCCAACCGTYTGACCTC R: GMYTGGTTGCGRATGGT	608	55

### Fish Infection Experiments

The *L. maculatus* weighing approximately 18 g were cultured in aerated ponds at the Zhuhai experimental base of the South China Sea fisheries Research Institute of the Chinese Academy of Fishery Sciences, Guangzhou, China. The water temperature was controlled at 28 ± 1°C. During the temporary feeding period, fish were fed a basal diet of 5% body weight at 7 am and 6 pm every day. After feeding for 15 min, the residual feed was removed to prevent the water from becoming contaminated and fish were temporarily reared for a week. Before the experiment, three fish were randomly selected for visceral bacteriological examination and gill parasite monitoring. *A. veronii* was transferred to NB medium at 5% and cultured for 10 h at 28°C and 200 rpm/min. The concentration of bacterial suspension was adjusted to 3.9 × 10^9^, 3.9 × 10^8^, 3.9 × 10^7^, 3.9 × 10^6^ and 3.9 × 10^5^ cfu/ml, respectively. Three hundred healthy *L. maculatus* were randomly selected. Eugenol was used to anesthetize the *L. maculatus* before infection. Intraperitoneal injection and intramuscular injection was carried out using 0.1 ml of the above different concentrations of the bacterial suspension. The control group received an intraperitoneal injection and intramuscular injection of an equal volume of 0.85% normal saline. Bacteria from the liver, spleen, and ascites fluid of experimentally infected fish were re-isolated. All protocols for experiments involving live animals conducted in this study were approved by the Animal Care and Use Committee of Zhongkai University of Agriculture and Engineering, Guangzhou, China.

### Histopathological Examination

Heart, liver, kidney and spleen tissue from the moribund fish after infection were fixed in 10% buffered formalin solution, dehydrated in ethanol, embedded in paraffin wax blocks and sectioned, then stained with hematoxylin and eosin for histopathological observation.

## Results

### Clinical Symptoms of Naturally Infected Fish

**Figure 1:**
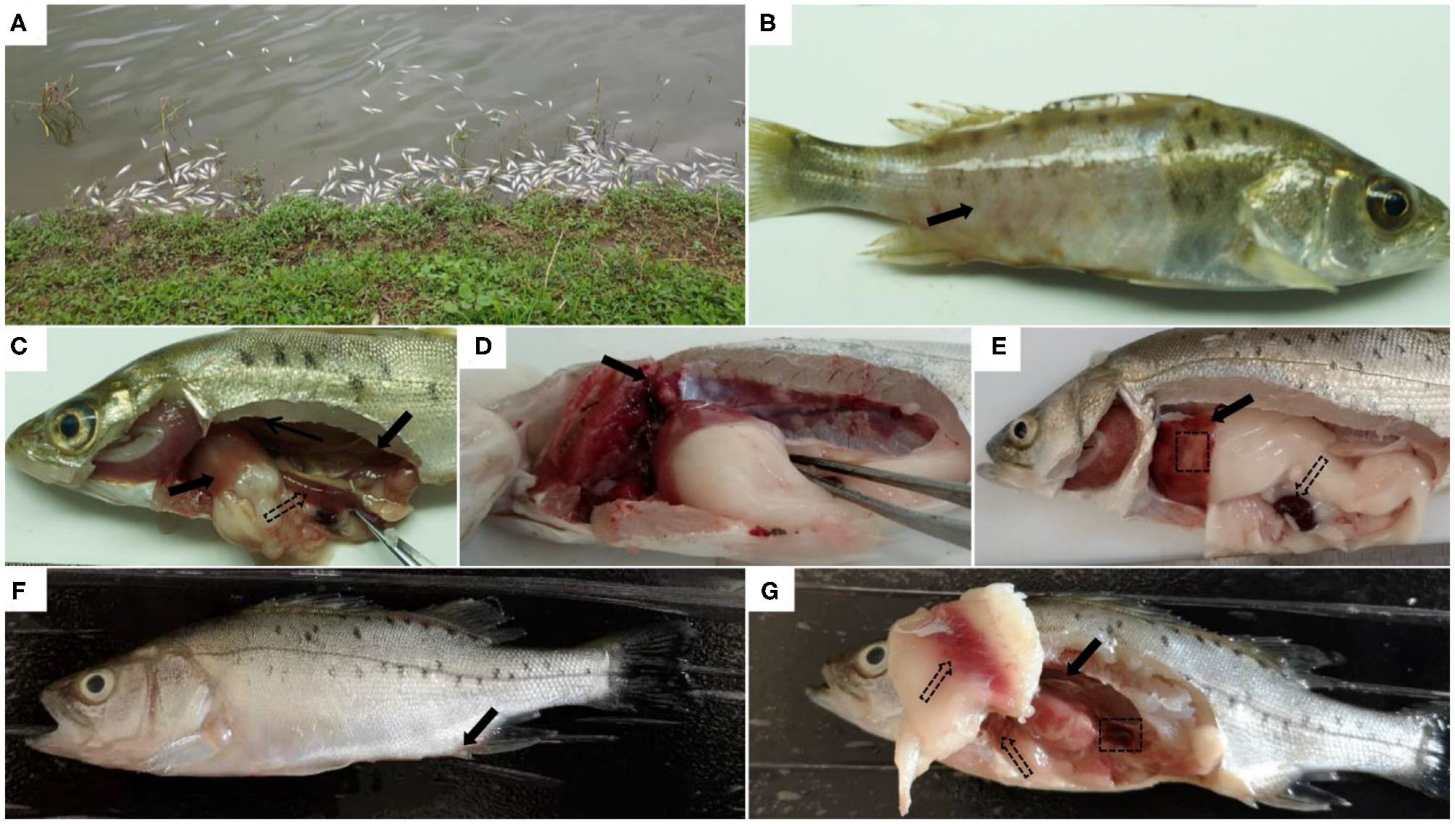
Clinical symptoms of natural or artificial infection of *Aeromonas veronii* in *Lateolabrax maculatus*. Acute death of *L. maculatus* in fish farms **(A)**. Obvious clinical symptoms of natural infection with *A.veronii* in diseased *L.maculatus*, such as ulceration on the body surface (solid arrow) **(B)**, hemorrhages in internal organs (solid arrows) and intestinal inflammation (hollow arrows) **(C)**, hemorrhage and swelling of the kidney (solid arrows) **(D)**, liver with both ischemia (black box), hemorrhage (solid arrow) and darkened spleen (hollow arrows) **(E)**. Clinical symptoms of *L.maculatus* artificially infected with *A.veronii*: the anal fin is slightly inflamed (solid arrow) **(F)**, slightly darkened spleen (black box), congestion in the abdominal wall and liver (hollow arrows), and hemorrhage and swelling in the kidney (solid arrows) **(G)**.

The *L. maculatus* naturally infected with *A. veronii* typically show acute death in aquaculture conditions. After 2–3 days of infection, fish begin to swim slowly leading to high mortality (Figure 1A). Some diseased fish exhibit large areas of ulceration on the body surface, limited to the surface of the skin (Figure 1B). After dissecting the fish, it was found that there were mild ascites in the abdomen. Hemorrhage of internal organs and intestinal inflammation were often observed (Figure 1C). Some fish showed typical clinical lesions, as well as swelling and hemorrhage of the kidney (Figure 1D), ischemia and hemorrhage in the liver, and darkening of the spleen (Figure 1E).

### Physiological and Biochemical Characteristics of the Bacteria

The strain 18BJ181 is a typical gram-negative bacterium isolated directly from diseased *L. maculatus*. Physiological and biochemical results in this study showed that 16 biochemical reactions of the strain 18BJ181 were positive, such as Ala-Phe-Pro arylamidase, L-Proline arylamidase, and sucrose, while 22 biochemical reactions were negative, such as H_2_S production and β-glucuronidase in a total of 38 biochemical reactions (Table 2). This species strain is distinguishable from the other three *Aeromonas* species [*A. veronii bv. veronii, A. hydrophila*, and *A. caviae*, (16]]. Due to the complexity of *Aeromonas* members and the limited number of biochemical reactions of Vitek 2, the test identified these isolates as *Aeromonas* species (probability > 99%), but failed to correctly differentiate them to the species level.

**Table 2:** Biochemical characterization of the strain 18BJ181 from naturally infected *Lateolabrax maculatus* in GuangDong province using Vitek 2 compact.

**Characteristics**	**18BJ181**	***Aeromonas veronii* ATCC9071**	***Aeromonas hydrophila* CGMCC1.2017**	***Aeromonas caviae* CGMCC1.1960**
Ala-Phe-Pro arylamidase	+	-	+	-
H_2_S production	-	-	+	-
β-glucoronidase	-	-	-	-
L-Proline arylamidase	+	+	+	+
Sucrose	+	+	+	+
L-Lactate alkalinization	-	-	+	+
Glycine arylamidase	+	-	-	+
O/129 resistance	+	+	+	+
β-Galactosidase	+	+	+	+
D-Maltose	+	+	+	+
Lipase	-	+	+	+
Ornithine decarboxylase	-	+	-	-
Glu-Gly-Arg-arylamidase	+	+	-	+
D-Mannitol	+	+	+	+
D-Trehalose	+	+	+	+
Succinate alkalinisation	+	+	+	+
L-Malate assimilation	-	-	-	+
D-Glucose	+	+	+	+
D-Mannose	+	+	+	-
Tyrosine arylamidase	+	+	+	+
Citrate	-	+	-	-
β-N-Acetyl-glucosaminidase	-	+	+	+
Ellman	+	+	+	+
D-Cellobiose	-	+	-	+
Courmarate	+	+	-	+
L-Lactate alkalinization	-	-	+	+

*The results of 12 biochemical reactions including adonitol, D-tagatose, L-pyrrolydonyl-arylamidase, glutamyl arylamidase pNA, lysine decarboxylase, L-arabitol, L-histidine assimilation, γ-glutamyl-transferase, β-xylosidase, urease, malonate and α-glucosidase of the strain 18BJ181 and reference Aeromonas were all negative (16)*.

### Phylogenetic Analyses of the 16S rRNA and *gyrB* Genes

**Figure 2:**
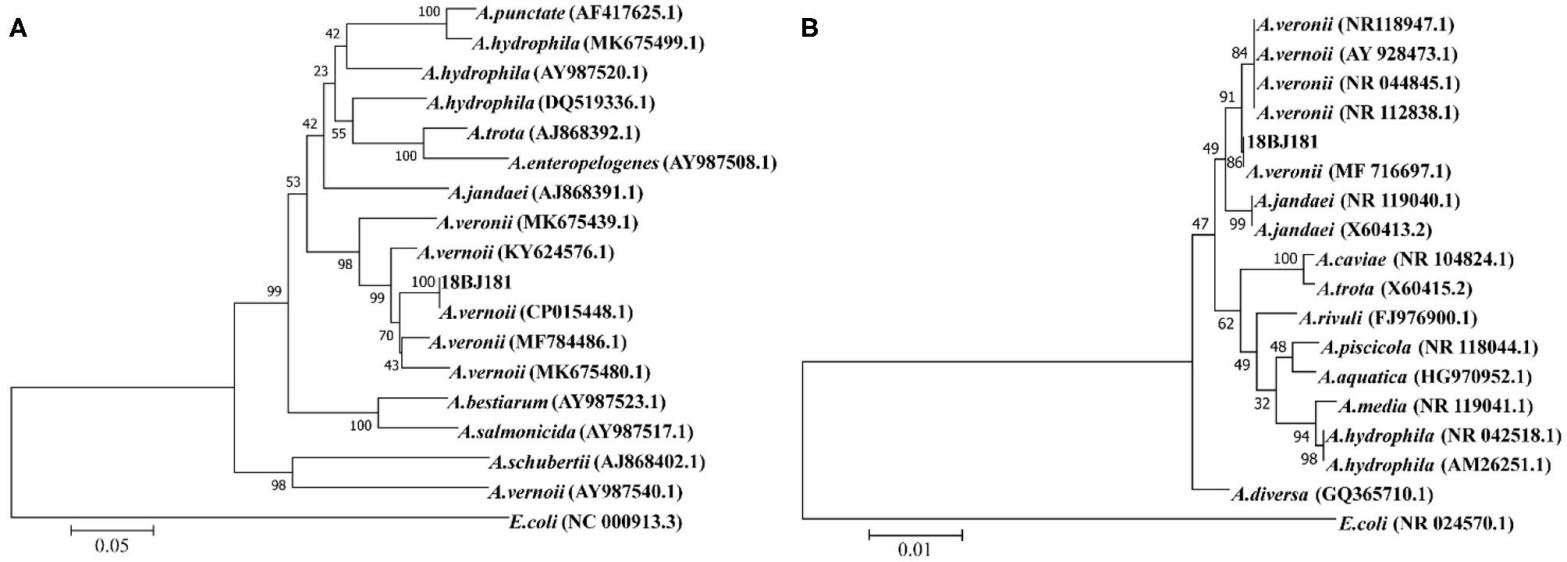
Neighbor-Joining phylogenetic tree generated based on 16S rRNA **(A)** *gyrB* **(B)** gene sequences of *Aeromonas veronii* isolates detected in the present study and the other *Aeromonas* spp. from Genbank. *Escherichia coli* was used as an outgroup species. Bootstrap values out of 1,000 repetitions were indicated above each branch.

The gene sequences of different *Aeromonas* were downloaded from the NCBI database and a phylogenetic tree was constructed. The 16S rRNA gene sequence of the strain 18BJ181 was 1,445 bp in length. The BLAST alignments showed that it was most similar to strain *A. vernoii* XG3-1-1 (MF716697.1). The *gyrB* gene sequence of the strain 18BJ181 was 1,045 bp in length. The BLAST alignments showed that it was most similar to strain *A. vernoii* CB51 (CP015448.1). Identities were approximately 99.81%. In addition, the results showed that the strain 18BJ181 was grouped with a cluster of known species of *A. veronii* strains according to phylogenetic trees established on the 16S rRNA sequence and *gyrB* sequence (Figure 2). Based on biochemical tests and phylogenesis through 16S rRNA and *gyrB* genes, the strain 18BJ181 was determined to be a member of *Aeromonas veronii*.

### Growth Characteristics of the Strain 18BJ181

**Figure 3:**
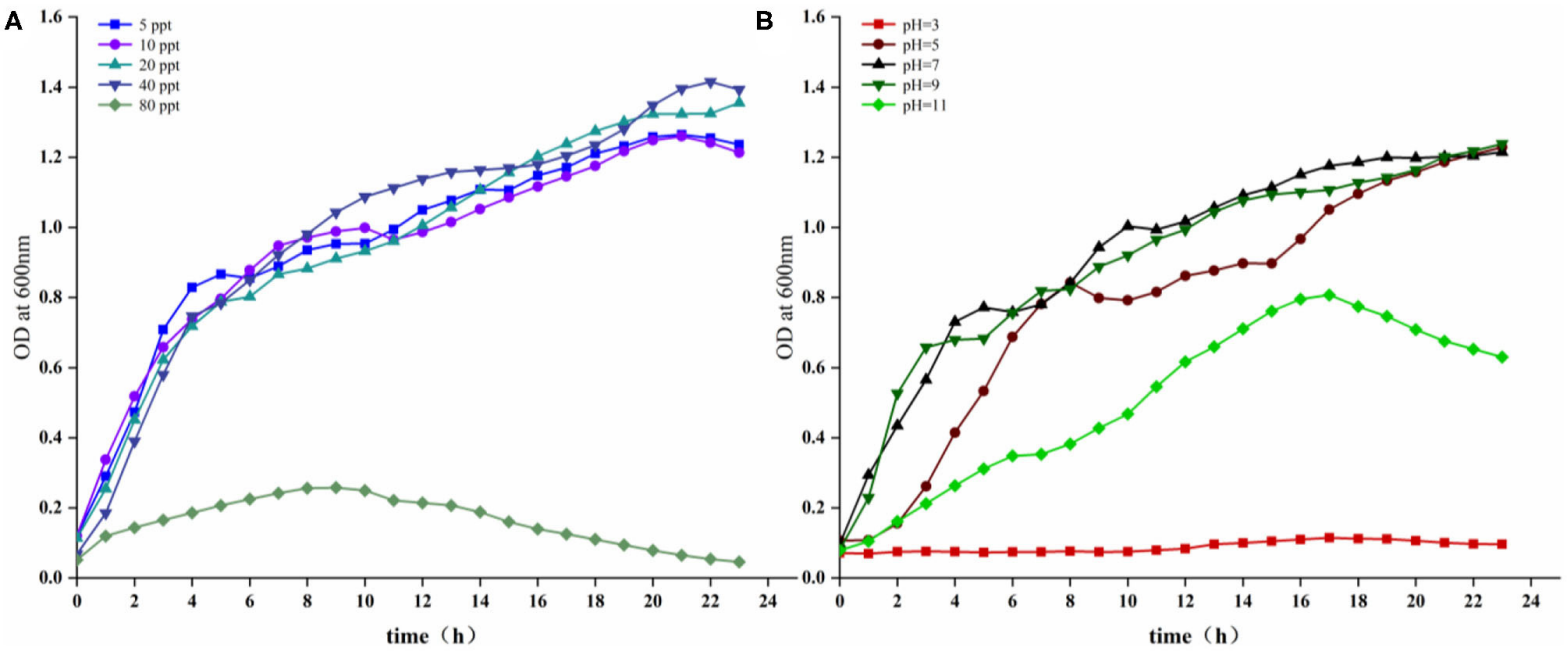
Growing characteristics of the strain 18BJ181. Growth under the conditions of different salinity **(A)**, different pH **(B)**.

Growth characteristics of the strain 18BJ181 were tested (Figure 3). As shown in Figure 3A, growth was similar at salinity concentrations of 5, 10, 20, and 40 ppt. Growth was improved at 20 and 40 ppt and significantly inhibited at a salinity of 80 ppt. In terms of pH, as shown in Figure 3B, the growth of this isolate was maximal at an optimum of pH 7. The latent phase of growth was somewhat extended at pH 11 and growth capacity was reduced. At pH 7 and 9, the growth of the strain 18BJ181 showed that trends were similar, and considerable final concentrations were obtained. Growth was halted at pH 3.

### Drug Sensitivity

The drug sensitivity results indicated that the strain 18BJ181 showed different sensitivities to 16 antibacterial drugs. Strain 18BJ181 was sensitive to 10 antibacterial drugs such as sulfamethoxazole, trimethoprim, and chloramphenicol, showed moderate sensitivity to erythromycin and rifampicin, and was resistant to amoxicillin, madinomycin, penicillin and sulfamethoxazole (Table 3).

**Table 3:** Antibiotic sensitivity of the strain 18BJ181.

**Antibiotics**	**Content (ug/disc)**	**Sensitivity**
Sulfamethoxazole and Trimethoprim	1.25	S
Amoxicillin	20	R
Chloramphenicol	30	S
Doxycycline	30	S
Erythromycin	15	M
Enrofloxacin	30	S
Florfenicol	30	S
Furazolidone	100	S
Gentamicin	10	S
Madinomycin	30	R
Neomycin	10	S
Norfloxacin	10	S
Oxytetracycline	30	S
Penicillin	10	R
Rifampicin	5	M
Sulfamethoxazole	300	R

*S, sensitive, M, moderately susceptible, R, resistant*.

### Virulence Factors

**Figure 4:**
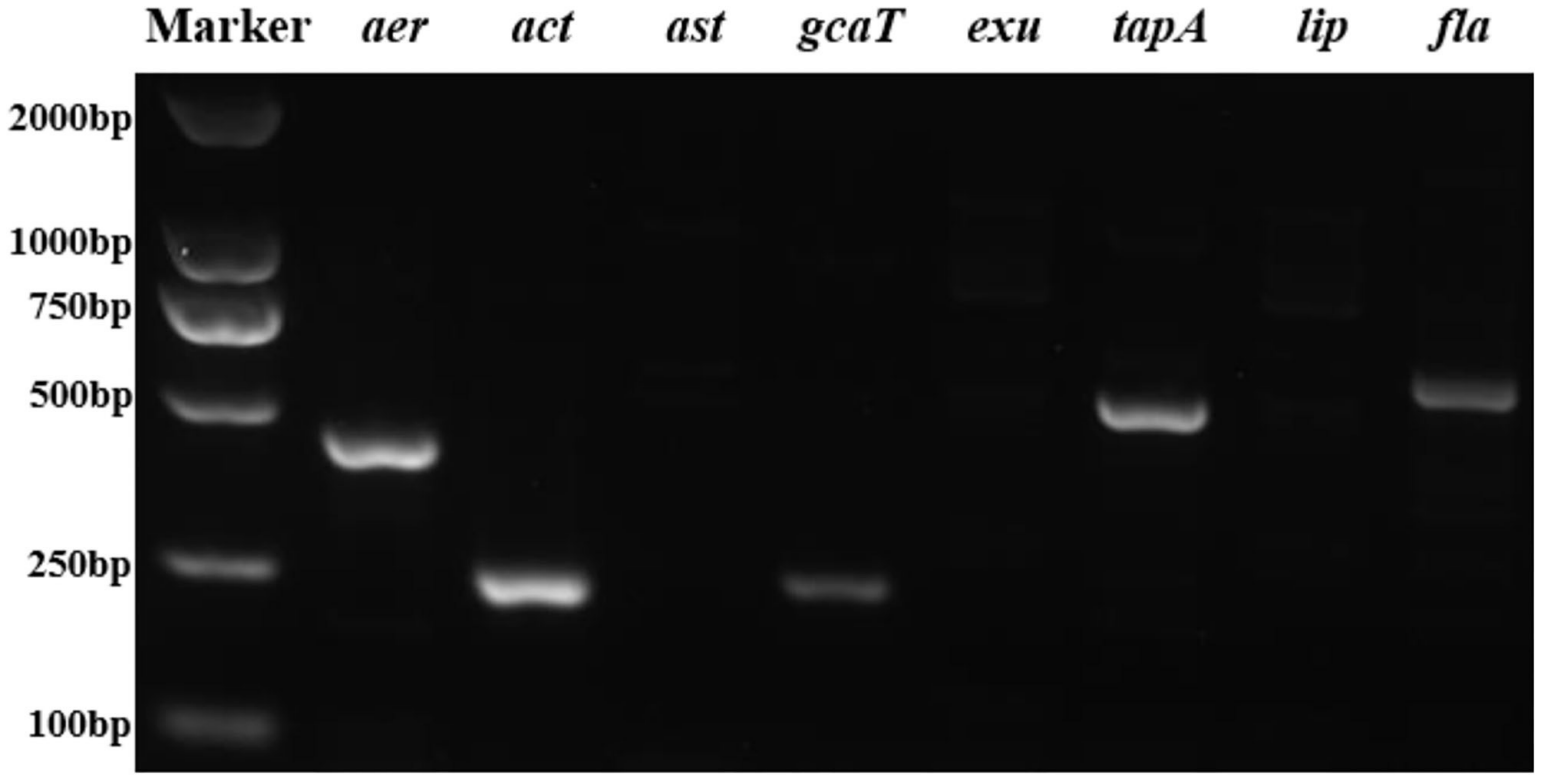
Agarose gel electrophoresis of eight virulent genes (*aer, act*, ast, *gcaT, exu, tapA, lip*, and *fla*) were present in the strain 18BJ181.

The PCR profiles of eight virulence genes screened in this study showed that five genes (*aer, act, gcaT*, tap*A*, and *fla*) was present in the strain 18BJ181 isolate (Figure 4).

### Experimental Infections

**Figure 5:**
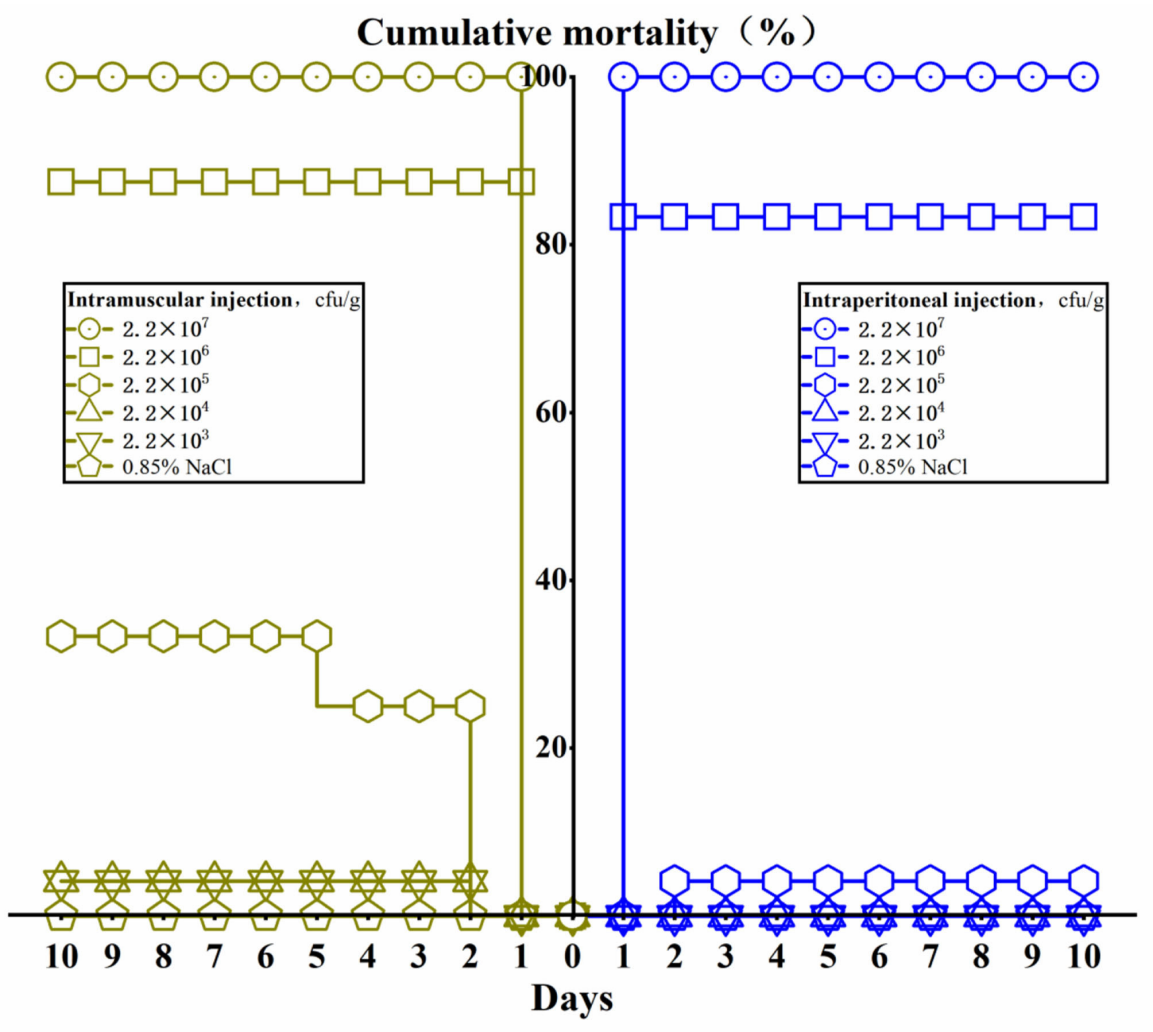
The cumulative mortality of *Lateolabrax maculatus* from different infection routes of *Aeromonas veronii* in 10 days.

*L. maculatus* can be infected successfully by intraperitoneal injection and intramuscular injection (Figure 5). The results showed that mortality of *L. maculatus* occurred within 24 h, and the number of deaths decreased significantly within a 48 h period. When the concentration of intraperitoneal injection and intramuscular injection was 2.2 × 10^7^ cfu/g, the mortality rate of the fish reached 100%. When the infection concentration was 2.2 × 10^6^ cfu/g, the mortality rate for intraperitoneal injection and intramuscular injection was 83.3 and 87.5%, respectively. Using an infection concentration of 2.2 × 10^5^ cfu/g, the mortality rate for intraperitoneal injection and intramuscular injection was 4 and 25%, respectively. When the infection concentrations were 2.2 × 10^4^ cfu/g and 2.2 × 10^3^ cfu/g, respectively, fish with intraperitoneal injection did not die and the mortality rate of fish infected by intramuscular injection was also very low, no more than 5%. The median lethal dosage (LD_50_) through intraperitoneal injection and intramuscular injection was calculated by Karber's method as 8.5 × 10^5^ and 4.2 × 10^5^ cfu/g, respectively.

### Pathological Analysis of Artificially Infected Fish

There were no obvious lesions on the body surface of *L. maculatus* that were artificially infected with the strain 18BJ181 isolate. The predominant symptoms were redness and swelling of the anal fin (Figure 1F). After dissection of the infected fish, it was observed that the ascites fluid in the abdominal cavity increased significantly. The abdominal wall and liver were congested. Kidney enlargement and hemorrhage were observed, and the spleen became darker in color (Figure 1G).

**Figure 6:**
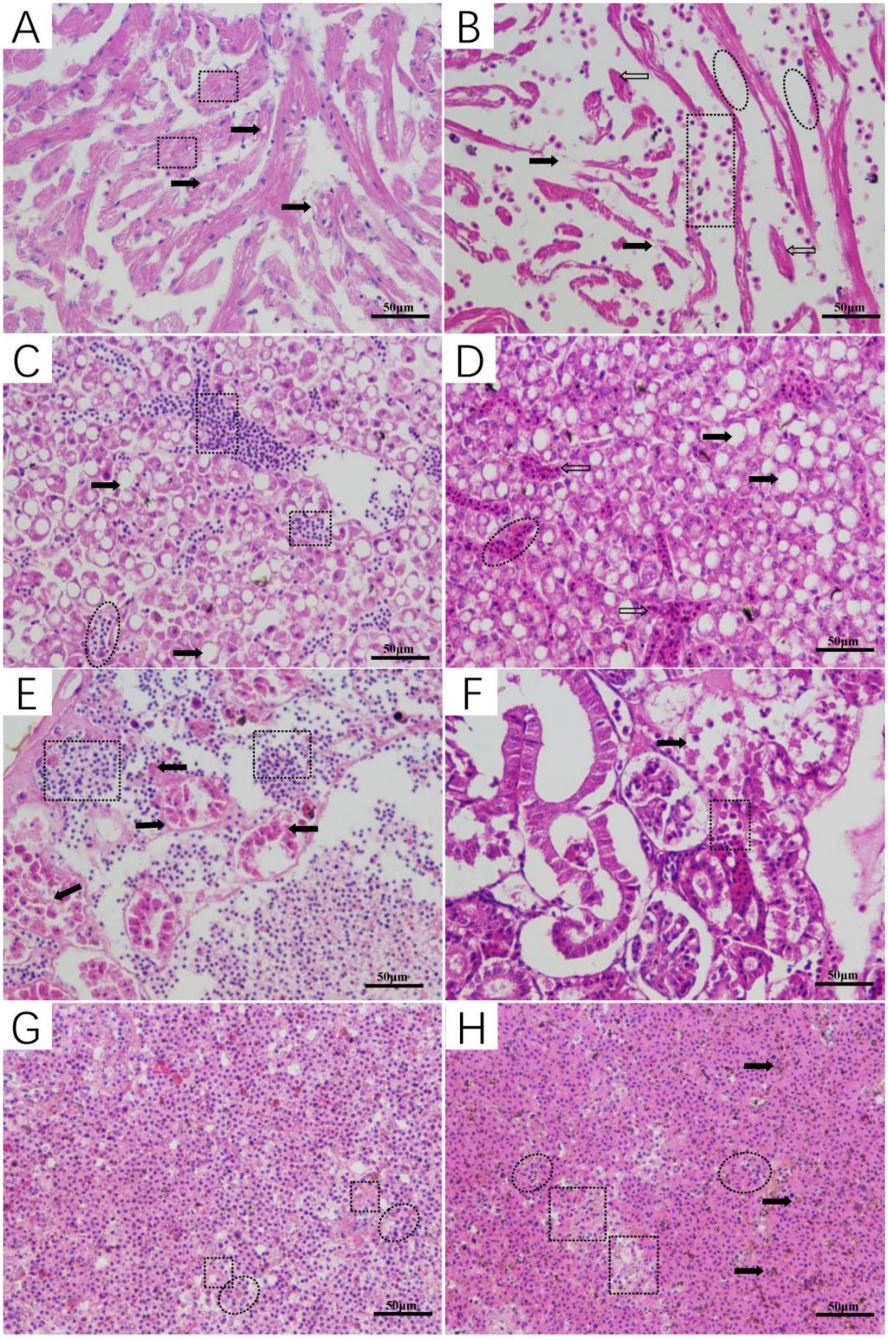
Histopathological changes of *Lateolabrax maculatus* with artificial infection of *Aeromonas veronii* (**A,C,E,G**: intraperitoneal injection, **B,D,F,H**: intramuscular injection). Cardiomyocyte vacuolization (solid arrows) with myofibrillar degeneration (rectangular) **(A)** and myocardial hemorrhage (rectangular) with myofibrillar edema (ovals), rupture (solid arrows) and necrosis (hollow arrows) **(B)**. Hepatocellular steatosis (solid arrows), congestion (ovals), hemorrhage (rectangular), and inflammation (hollow arrows) in the liver **(C,D)**. Kidney with marked necrosis and desquamation of the renal tubular epithelial lining leaving the basement membrane (solid arrows) notice the hemorrhage in the interstitial tissue (rectangular) **(E,F)**. Moderate splenitis with numerous lymphocytes and macrophages aggregated around ellipsoids (ovals) in addition to an accumulation of proteinaceous substance (rectangular) **(G)**. Splenitis with diffuse fibrinoid necrosis of ellipsoids (rectangular), aggregation of inflammatory cells (ovals), and hemozoin accumulation (solid arrows) **(H)**.

The histopathology of *L. maculatus* infected by intraperitoneal injection of *A. veronii* was as follows: cardiomyocyte vacuolization with myofibrillar degeneration was observed in the heart (Figure 6A), the liver showed different levels of hepatocellular steatosis, congestion, and hemorrhage (Figure 6C), the renal tubular epithelial lining was severely necrotic, and detached from the basement membrane, and interstitial tissue hemorrhage was evident (Figure 6E), and moderate splenitis was observed with numerous lymphocytes and macrophages aggregated around ellipsoids, in addition to an accumulation of a proteinaceous substance accumulated in the spleen (Figure 6G). The histopathological changes of *L. maculatus* infected by intramuscular injection were as follows: myocardial hemorrhage with myofibrillar edema, rupture, and necrosis was observed in the heart (Figure 6B), the liver had histopathological changes such as hepatocellular steatosis, congestion, and inflammation (Figure 6D), necrosis and desquamation of the renal tubular epithelial lining and interstitial tissue hemorrhage were relatively mild in the kidney (Figure 6F), and the spleen showed pathological changes such as splenitis with diffuse fibrinoid necrosis of ellipsoids, aggregation of inflammatory cells, and accumulation of hemozoin (Figure 6H).

## Discussion

*A. veronii* is widely distributed and can be isolated from diseased aquatic animals and aquatic environments (17–19) in various countries such as Poland (20), Mexico (21), and Japan (22). The pathogen can cause human biliary sepsis and diarrhea in clinical practice (23, 24). The physiological and biochemical characteristics of the strain 18BJ181, such as L-Proline arylamidase, β-Galactosidase and O/129 resistance, were positive with *A. veronii* ATCC9071. Due to the wide variety of *Aeromonas* species and the limited number of reactions of Vitek 2, the molecular method is required to distinguish the species level of *Aeromonas* (16). 16S rRNA is one of the most commonly used molecular biological detection methods for identifying bacteria (25) and the *gyrB* gene has certain advantages for the identification of *Aeromonas* species (26). In this study, BLAST alignments showed that both 16S rRNA and *gyrB* gene sequences of the strain 18BJ181 shared the highest identities with those of other known *A. veronii* strains. The phylogenetic trees built based on the sequences of the two genes showed the strain 18BJ181 clustered with *A. veronii* strains. For the identification of *A. veronii*, it is important to use multiple molecular markers for phylogenetic analysis. If this is not possible, another approach is to conduct genomic sequence analysis for a specific strain, then combine them with epidemiological evidence for a more comprehensive analysis. Using these methods, it is possible to provide new insights into the complex evolutionary history of *A. veronii*.

*A. veronii* has a variety of hosts and can live in aquatic animals and in the environment, which may bring harm to aquaculture in the future. Bacterial resistance to antibiotics affects the health of animals, the environment, and humans (27). Previous research results showed that *A. veronii* was resistant to antibacterial drugs, including ampicillin, amoxicillin and oxacillin (28–31). In this study, we confirmed part of these results, specifically that strain 18BJ181 is resistant to amoxicillin and penicillin. Over time, *A. veronii* has developed a certain resistance to sulfamethoxazole. The results of drug sensitivity testing on the strain 18BJ181 isolate in this study provides a reference for when antimicrobials may be a potential treatment for *A. veronii*.

The pathogenicity of *A. veronii* is related to the expression of virulence factors (32). *Aer* is a cytotoxic pore-forming enterotoxin, which is one of the most important and abundant virulence factors of *A. veronii*. Isolates from all tissue necrosis and ulcerations in fish of *A. veronii* induced aeromonas septicemia and bacterial haemorrhagic septicemia were positive for the *aer* gene. The *aer*-positive isolates from crap fish and catfish were 52.9% and 82.4%, respectively. Fish injected with *A. veronii* exhibited significantly higher mortality than carp fish [*P* < 0.05, (33)]. Using an infection route via intestine, a comparison of *A. veronii* strain Hm091 with *A. hydrophila* showed that different expression and activity of the *aer* gene was the key factor that caused the difference in virulence between the two species (8). We detected the *aer* gene from *A. veronii*, which may have indicate a connection with its strong pathogenicity. *Act* gene plays an important role in *A. hydrophila* and can significantly reduce capacity to evoke fluid secretion (34). Glycerophospholipid-cholesterol acyltransferase and lipases paly a common role in the pathogenicity of *Aeromonas* spp and, together, are secreted into the environment through the secretion system (5, 35). *TapA* of *Aeromonas salmonicida* participates in the process of infecting Atlantic salmon (36). The *fla* is involved in the formation of *Aeromonas* spp biofilms and has potential pathogenicity (37). In this study, virulence genes *aer, act, gcaT, tap*A and *fla* were detected from strain 18BJ181, but the more complex mechanism of toxicity action of *A. veronii* still needs to be further explored.

In this study, the time of death of *L. maculatus* was concentrated within 24 h after *L. maculatus* was infected by *A. veronii* infection. This finding is consistent with the death of *A. veronii* infection in *Carassius auratus gibelio* and *Xiphophorus helleri* (12, 38). This suggests that the rapid death of the host will accelerate the spread of the disease. The LD_50_ in the intramuscular injection group was slightly lower than in the intraperitoneal injection group, but the mortality of both showed a “cliff” decline. *Z*ebrafish is also a susceptible host of *A. veronii*. It has been found that this fish can be successfully infected with *A. veronii* through different infection routes (8). Likewise, *A. hydrophila* showed muscle liquefaction and furuncle lesions in infected rainbow trout (*Oncorhynchus mykiss*) after intramuscular injection (39). The difference in the expression and activity of the *aer* gene between *A. veronii* and *A. hydrophila* resulted in *A. veronii* being slightly more virulent than *A. hydrophila*, and this in turn activates the activity of the *aer* gene and functions in enhancing the adhesion ability of bacteria in host cells (8). In addition, the quorum-sensing system plays a key role in the infection of *A. veronii* and is a major metabolic regulator in *A. veronii* and participating in sturgeon spoilage (40). This is related to the quorum-sensing system of *A. veronii* and *A. hydrophila* in regulating virulence in catfish (41). The virulence regulation effect of *Aeromonas* on burbot (*Lota Lota*) was also regulated by quorum sensing (42). N-acyl homoserine lactone (AHL) mediated by the quorum-sensing mechanisms may be involved in regulating the type VI secretion system (T6SS), metalloprotease production, biofilm formation, and virulence of *A. veronii* (43). As previously reported, AHL molecules are not only involved in bacterial virulence regulation but also interact with several eukaryotic cells and play function in the immuno-modulation of the host response in *Pseudomonas aeruginosa* (44). However, the exact mechanism of action is unclear. *L. maculatus* infected with *A. veronii* died acutely in a short period of time and correlated with the concentration of infection. This indicates that it may be closely related to the quorum-sensing system, but the details still need to be further studied.

*Aeromonas* can cause an infection characterized by septicemia and spread within 1 hpi through the organs, affecting irreversible lesions to the liver, kidney and spleen. The pathogen was detected in the spleen at 3 hpi, and the greatest amounts of *Aeromonas* and lesions were observed at 6 and 9 hpi in all evaluated organs (*p* < 0.05) (45). *Aeromonas* infection of fish also cause skin ulcers, intra-abdominal hemorrhage, and other clinical symptoms. Interestingly, there are a variety of symptoms caused by different types of *Aeromonas* infections (15). For example, the clinical signs of bacterial septicemia in catfish caused by *A. veronii* were pale gills, slight abdominal distension, and swollen and inflamed vents (46). When *A. veronii* infect Sheatfish, petechial skin hemorrhages appear, as well as ascitic distension of the abdomen, redness, and swelling of the anus (47). *A. veronii* can cause histopathological changes such as hemorrhage, congestion, and ulcers in different organs (6). Some cases of *A. veronii* infected fish showed degenerative histopathological changes such as cellular vacuolation, intravascular congestion, and cell necrosis (3). In this study, the histopathological results were similar to those of previous studies. For example, in some internal organs there were histopathological changes, especially the necrosis of the renal tubular epithelial lining and interstitial tissue hemorrhage. These symptoms provided direct evidence of the cause of death in *L. maculatus*.

In conclusion, this is the first study to report a case of *A. veronii* infection in *L. maculatus* in China. Clinical symptoms of naturally infected fish were acute death, ulceration on the body surface, congestion, and hemorrhages in internal organs. *A. veronii* is resistant to amoxicillin, madinomycin, and other antimicrobial agents and is suitable for survival in the environment. Using artificial infection, *A. veronii* can infect *L. maculatus* through intraperitoneal injection and intramuscular injection, causing abdominal hemorrhage and congestion, as well as pathological damage to the heart, liver, kidney, and spleen including degeneration, necrosis, and hemorrhage. As a newly reported bacterial disease in *L. maculatus*, this report sheds new light on understanding the characteristics of *A. veronii* regarding host pathogenicity. Further epidemiological investigation and retrospective studies are needed, as well as further exploration of the relationship between pathogenic bacteria and the host.

## Data Availability Statement

The datasets presented in this study can be found in online repositories. The names of the repository/repositories and accession number(s) can be found in the GenBank: MW362188 for *Aeromonas veronii* strain 18BJ181 16S ribosomal RNA gene and MW371213 for *Aeromonas veronii* strain 18BJ181 *gyrB* gene partial cds, respectively.

## Ethics Statement

The animal study was reviewed and approved by the Animal Care and Use Committee of Zhongkai University of Agriculture and Engineering.

## Author Contributions

YS and BW conceived and designed as well as analyzed the experiments. BW performed all the experiments and wrote the paper. CM completed the Antimicrobial resistance test. JH, YL, and QG assisted in laboratory experiments and data analysis. JF, BJ, and YS participated in discussions and revisions and critically examines the final uploaded manuscripts. All authors read and agreed to the final version of the manuscript.

## Conflict of Interest

The authors declare that the research was conducted in the absence of any commercial or financial relationships that could be construed as a potential conflict of interest.
